# Structure Determination and Anti-Inflammatory  Activity of some Purine Complexes

**DOI:** 10.1155/MBD.1995.13

**Published:** 1995

**Authors:** Satwinder S. Marwaha, Jasjest Kaur, Gurvinder S. Sodhi

**Affiliations:** 1 Department of Biotechnology Punjabi University Patiala 147002 India; 2 Department of Chemistry University of Delhi Delhi 110007 India; 3 Department of Chemistry S.G.T.B. Khalsa College University of Delhi Delhi 110007 India

## Abstract

Organomercury(II)-purine derivatives of the type, p-MeOC_6_H_4_HgL^1^ (I), p-NO_2_C_6_H_4_HgCl(L^2^)(II), p-MeC_6_H_4_HgCl(L_3_)(III) and p-NO_2_C_6_H_4_HgCl(L^3^)(IV) [ HL^1^ = theophylline, L^2^ = theobromine, L^3^ = caffeine] have been synthesised and characterised on the basis of spectral studies (IR, UV, ^1^H & ^13^C NMR). The complexes have been screened for anti-inflammatory activity.


### STRUCTURE DETERMINATION AND ANTI-INFLAMMATORY ACTIVITY OF SOME PURINE COMPLEXES

Satwinder S. Marwaha[1], Jasjest Kaur[2] and Gurvinder S. Sodhi*[3]

[1] Department of Biotechnology, Punjabi University, Patiala-147002, India
[2] Department of Chemistry, University of Delhi, Delhi-110007, India
[3] Department of Chemistry, S.G.T.B. Khalsa College, University of Delhi, Delhi-110007, India

### ABSTRACT

Organomercury(II)-purine derivatives of the type, p-$MeOC_6H_4HgL^1$ (I), p-$NO_2C_6H_4HgCl(L^2)$ (II), p-$MeC_6H_4HgCl(L^3)$(III) and p-$NO_2C_6H_4HgCl(L^3)$ (IV) [$HL^1$ = theophylline, $L^2$ = theobromine, $L^3$ = caffeine] have been synthesised and characterised on the basis of spectral studies (IR, UV, $^1$H & $^{13}$C NMR). The complexes have been screened for anti-inflammatory activity.

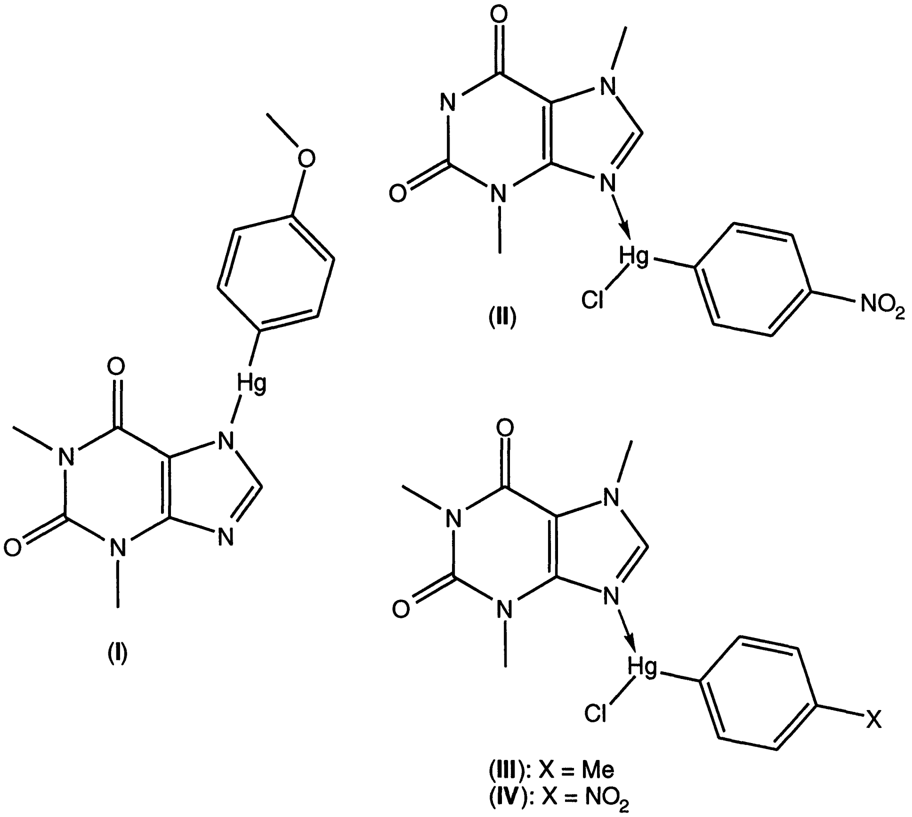

[1] From Ph.D. thesis of J.K.

* Present address: Department of Chemistry, S.G.T.B. Khalsa College, University of Delhi, Delhi 110007, India

** Author to whom correspondence should be addressed

### INTRODUCTION

The purines constitute an important class of anti-inflammatory agents. Important members of this class include theophylline, theobromine and caffeine. They owe their anti-inflammatory action to their capacity to affect changes in the basic cellular functions. These compounds act as competitive inhibitors of phosphodiesterase, an enzyme which inactivates cyclic 3', 5'-adenosine monophosphate (3',5'-c AMP)[1]. The enzyme inhibition activates 3',5'-c AMP, which consequently stimulates glycoganolysis and promotes cellular processes. It has also been postulated that the purine drugs act by reducing the binding of calcium in the membrane and myoplasm of the cell, thereby affecting the contractility of the muscles[1].

The interest in the metal complexes of purines has arisen for the following reasons: Firstly, as a result of antitumor activity of some metal complexes, considerable interest has been shown in the design of model complexes involving purines which could mimic the interaction of metal ions with DNA[2-4]. Secondly, a few metal-purine complexes have shown significant anti-inflammatory activity[5]. Thirdly, the investigation of mercury-purine complexes is of particular value, since the known toxic effects of the organomercury compounds, which are attributed to the formation of mercury-sulphur bonds with sulphydryl functions in amino acids and proteins, may also be due, in part, to their interaction with nucleic acid constituents[6]. Therefore, we have carried out the synthesis of a few organomercury(II)-purine complexes and have determined their structure and anti-inflammatory activity.

### EXPERIMENTAL

The following instruments, were used: Elico conductivity bridge model CM-82; Shimadzu spectrophotometer, IR-435; Perkin-Elmer UV-VIS spectrophotometer, model 554; Jeol FX-200 FT-NMR spectrometer. Theophylline, theobromine and caffeine were purchased from Sigma Chemical Co., U.S.A. and used without further purification. The arylmercury(II) chlorides, p-$MeC_6H_4HgCl$, p-$MeOC_6H_4HgCl$ and p-$NO_2C_6H_4HgCl$ were synthesised by the method of Nesmeyanov *et al*[7].

**Preparation of Complexes.** The theophylline complex, p-$MeOC_6H_4HgL^1$ was prepared by slowly adding a solution of p-$MeOC_6H_4HgCl$ (0.01 mol) in 25 ml DMF to a stirred solution of theophylline (0.01 mol) in 25 ml DMF at a pH of 8-9. The pH of the solution was set in the desired range by adding a few drops of 0.1M sodium bicarbonate solution. The contents were stirred for 6h at 50°C and filtered. The filtrate was slowly poured over crushed ice and stirred vigorously. The precipitates so obtained were washed successively with hot water and benzene. The resulting product was dried and recrystallized from THF. The theobromine complex, p-$NO_2C_6H_4HgCl(L^2)$ and the caffeine complexes, p-$XC_6H_4HgCl(L^3)$ (X = Me, $NO_2$) were synthesised from p-$XC_6H_4HgCl$ and the respective purines, by following a similar series of operations, except that the reaction was conducted in a neutral medium.

Some physico-chemical characteristics of the complexes are as follows:

(I) Dec. temp., 133°C.
Anal. Found (Calc.), %: Hg, 41.31 (41.21); C; 34.60 (34.53); N, 11.45 (11.51); H, 2.93 (2.87).
IR (nujol), $cm^{-1}$: $\gamma$ (c=0), 1630; $\gamma$ (c=c), 1580; $\gamma$ (c=N), 1530.
UV (DMF), nm (log $\varepsilon$) : 272 (7.0); 240 (3.1).
$^1H$ NMR ($d^6$-dmso), $\delta$ : 3.28 (s,3H,1-Me); 3.48 (s, 3H, 3-Me); 7.10-8.00 (m, 1H, H(8) + 4H(phenyl)); 3.75 (s, 3H, 4'-MeO).
$^{13}C$ NMR ($d^6$-dmso), ppm: Theophyllinato, C(2), 156.8; C(3), 148.5; C(5), 106.3; C(6), 160.3; C(8), 140.1; 1-Me, 27.3; 3-Me, 30.1; Phenyl, C(1'), 148.8; C(2',6'), 138.0; C(3', 5'), 122.0; C(4'), 151.1; 4'-MeO, 20.8.

(II) Dec. temp., 170°C

Anal. Found (Calc.), %: Hg, 37.32 (37.26); C, 28.91 (28.99); N, 13.06 (13.01); Cl, 6.65 (6.59); H, 2.26 (2.23).

IR (nujol) $cm^{-1}$: γ (c=0), 1640; γ (c=c), 1590; γ (c=N), 1520; γ (Hg-Cl), 370.

UV (DMF), nm (log ε) : 270 (6.2); 240 (3.2).

$^1H$ NMR ($d^6$-dmso), δ : 3.40 (t, 3H ,J1.8Hz,1-Me); 3.92 (s, 3H, 3-Me); 8.20 (s, 1H, H(8)); 7.00-8.05 (m, 4H (phenyl)).

$^{13}C$ NMR ($d^6$-dmso), ppm: Theobromine, C(2), 152.1; C(4), 148.5; C(5), 107.3; C(6), 160.2; C(8), 134.5; 3-Me, 30.1; 7-Me, 28.5; Phenyl, C(1'), 149.0; C(2',6'), 142.0; C(3', 5'), 124.5; C(4'), 135.3.

(III) Dec. temp., 212°C.

Anal. Found (Calc.), %: Hg, 38.58 (38.48); C, 30.49 (30.43); N, 10.71 (10.74); Cl, 6.90 (6.81); H, 2.46 (2.53).

IR (nujol) $cm^{-1}$: γ (c=0), 1645; γ (c=c), 1590; γ (c=N), 1510; γ (Hg-Cl), 370.

UV (DMF), nm (log ε) : 274 (5.5); 245 (3.0).

$^1H$ NMR ($d^6$-dmso), δ : 3.90 (s,3H,1-Me); 3.40 (s, 3H, 3-Me); 3.22 (s, 3H,7-Me); 8.20 (s, 1H, H(8)); 7.00-7.85 (m, 4H (phenyl)); 2.38 (s, 3H, 4'-Me).

$^{13}C$ NMR ($d^6$-dmso), ppm: Caffeine, C(2), 152.2; C(4), 148.5; C(5), 106.8; C(6), 161.1; C(8), 134.5; 1-Me, 26.8; 3-Me, 30.1; 7-Me, 28.5; Phenyl, C(1'), 147.3; C(2',6'), 151.0; C(3', 5'), 135.3; C(4'), 156.5; 4'-Me, 21.5.

(IV) Dec. Temp., 235°C

Anal. Found (Calc.), %: Hg, 36.30 (36.32); C, 30.47 (30.43); N, 12.73 (12.68); Cl, 6.49 (6.43); H, 2.57 (2.53).

IR (nujol) $cm^{-1}$: γ (c=0), 1650; γ (c=c), 1590; γ (c=N), 1505; γ (Hg-Cl), 370.

UV (DMF), nm (log ε) : 273 (5.6); 244 (3.0).

$^1H$ NMR ($d^6$-dmso), δ : 3.90 (s,3H,1-Me); 3.42 (s, 3H, 3-Me); 3.18 (s, 3H,7-Me); 8.20 (s, 1H, H(8)); 7.05-8.05 (m, 4H (phenyl)).

$^{13}C$ NMR ($d^6$-dmso), ppm: Caffeine, C(2), 152.0; C(4), 148.3; C(5), 107.3; C(6), 160.8; C(8), 134.4; 1-Me, 26.6; 3-Me, 30.0; 7-Me, 28.5; Phenyl, C(1'), 149.0; C(2',6'), 141.8; C(3', 5'), 124.4; C(4'), 135.5.

**Evaluation of Anti-Inflammatory Activity.** The method of Lakdawala *et al.*[8] based on carrageenan-induced paw edema in rats was used to evaluate anti-inflammatory activity of the complexes. It determines the ability of the test compound to inhibit the edema produced by carrageenan in the hind paw of the rats. Indomethacin was used as a reference.

Group of six rats (Charles Foster variety) were fasted overnight prior to the initiation of he experiment. Hind paw edema was induced by subcutaneous injection of carrageenan solution (0.1 ml) into the left hind paw of the rats. The right hind paw was injected with carboxymethylcellulose (CMC, 10 ml $Kg^{-1}$), which serves as a control. A suspension of the test compounds (100 mg $Kg^{-1}$) was prepared in aqueous gum tragacanth. The test compounds were administered orally. The volumes of both the hind paws were determined before and 3 h after the injection of carrageenan, with the help of mercury plathysmograph attached to Digitlog Recorder through Statham Pressure Transducer (p230b). Percent inhibition of increase in paw volume was calculated.

### RESULTS AND DISCUSSION

Satisfactory results of elemental analysis and spectral studies reveal that the complexes are of good purity. This is also supported by TLC. The complexes are soluble in THF, DMSO and acetone. Conductance measurements in $10^{-3}$M solution are of the order of 0.50 $ohm^{-1}$ $cm^2$ $mol^{-1}$, indicating that the complexes are non-electrolytes.

**Infrared Spectra.** Theophylline may chelate to the metal ion through N(7) and exocyclic oxygen at C(6) or may be bound to the metal ion through N(7) alone[3,9]. However, it is not possible to distinguish between these two binding modes on the basis of IR spectra for the balanced N7/O6 chelation compared with unidentate N7 binding introduces no changes in the 1500–1750 $cm^{-1}$ region[10].

For example, in the mercury-theophylline complexes reported by Norris *et al.*[9], the $\gamma$ (c=0) stretching frequency in free theophylline absorbed at 1680 and 1720 $cm^{-1}$, while in the metal complexes it was shifted to 1630–1665 $cm^{-1}$ although the crystal structure data ruled out the involvement in complexation of the carbonyl group at C(6). In the present theophylline complex too, the band attributed to the $\gamma$ (c=0) stretching frequency was lowered from 1660 $cm^{-1}$ to 1630 $cm^{-1}$. This shift, however, cannot be attributed to the interaction of the carbonyl at C(6) with mercury (II) ion, since the UV and $^{13}C$ NMR data indicated the non-chelating behaviour of theophylline. In the case of theobromine and caffeine complexes, the $\gamma$ (c=0) stretching frequency remained unaltered on complexation. Substantial shift from *ca.* 1550 $cm^{-1}$ to *ca.* 1520 $cm^{-1}$ were observed for the purine ring modes[10].

**UV Spectra.** The UV spectra of theophylline, theobromine and caffeine showed an absorption band at 270 nm (log $\varepsilon$ 7.5), 274 nm (log $\varepsilon$ 6.2) and 272 nm (log $\varepsilon$ 5.8), respectively due to the $\pi$ - $\pi^*$ transition of the chromophoric carbonyl group[11]. In the case of complexes, this absorption occurred at *ca.* 272 nm. Since no significant shift was observed in the $\lambda_{max}$ value, the possibility of bonding through carbonyl group was ruled out.

**$^1H$ NMR Spectra.** In the $^1H$ NMR spectra, the theophyllinato group showed no significant shift on complexation. However in theobromine and caffeine, the signal due to H(8), which absorbed at *ca.* $\delta$ 8.00 (s, 1H), was shifted to $\delta$ 8.20 on complexation; the downfield shift was attributed to the involvement of N(9) in complexation.

**$^{13}C$ NMR Spectra**. In the $^{13}C$ NMR spectra, the signal at *ca.* 160 ppm in case of free ligands was attributed to the C(6) carbonyl. Since no significant shift was observed on complexation, it was concluded that the carbonyl at C(6) was not coordinated to the mercury (II) ion[4]. However, the resonance signal due to C(8), which appeared at 140.1 ppm in theophylline , was shifted to 143 ppm in complex (I), supporting the assertion that complexation involved deprotonation at N(7). Similarly, in theobromine and caffeine the signal due to C(8) absorbed at *ca.* 134.5. In complexes (II-IV), this signal absorbed at *ca.* 138 ppm, supporting the involvement of N(9) in complexation.

**Anti-Inflammatory Activity**. The results of anti-inflammatory activity, presented in Table-1, reveal that at an oral dose of 100 mg $kg^{-1}$, the test compounds, (I), (II), (III) and (IV), produced 22, 58, 31 and 61 percent inhibition, respectively.

**TABLE I. ANTI-INFLAMMATORY ACTIVITY**

| Compound | Dose (mg $Kg^{-1}$ PO) | Edema (ml) | Inhibition (%) |
|---|---|---|---|
| CMC (control) | 10 | 0.88 | -- |
| (I) | 100 | 0.30 | 22 |
| (II) | 100 | 0.37 | 58 |
| (III) | 100 | 0.30 | 31 |
| (IV) | 100 | 0.34 | 61 |
| Indomethacin (reference) | 5 | 0.34 | 61 |

The nonsteroidal anti-inflammatory drugs generally possess an aromatic group and/or heteroaromatic rings (such as purine units) in their structure. From the point of view of the degree of anti-inflammatory action, two structural aspects are important. Firstly, the presence of chlorine, fluorine or pseudohalogen equivalents are generally activity enhancing. Secondly, ionic substituents, such as a nitro group on the aromatic ring of the molecule also enhance the activity[13]. The presence of such groups enables the drug to interact with the bi-layer cell membrane[14].

In the present study too, the variation in anti-inflammatory activity followed the above criteria. The complexes, (II) and (IV), which had both a chloro group and a nitro substituent in their structures, resulted in a significant inhibition of edema. Next in the anti-inflammatory action was the compound (III) which had a chloro group but no ionic substituent. The complex (I) which had neither a chloro group, nor an ionic substituent was least active of all the compounds considered in this investigation.

### CONCLUSION

Although the concentration of test samples was different from that of indomethacin (reference drug), it was possible to make a comparative assessment of the anti-inflammatory activity of the compounds and also to study the variation of biological activity vis-a-vis the nature of substituents on the complexes.

### ACKNOWLEDGEMENT

We thank the Council of Scientific and Industrial Research, New Delhi, for the award of a senior research fellowship to one of us (J.K.).

### REFERENCES

1 A. Grollman and E.F. Grollman, *Pharmacology and Therapeutics*, Lea and Febiger, Philadelphia, 1970, p. 192.
2 D.M.L. Goodgame, I. Jeeves, F.L. Phillips and A.C. Skapski, *Biochim. Biophys. Acta, 378* (1975) 153.
3 D.J. Hodgson, *Prog. Inorg. Chem., 23* (1977) 211.
4 B. Lippert, *Prog. Inorg. Chem., 37* (1989) 1.
5 S. Bhatia, N.K. Kaushik and G.S. Sodhi, *J. Chem. Research*, (S) (1987) 186; (M) (1987) 1519.
6 J.J. Mulvihill, *Science, 176* (1972) 132.
7 A.N. Nesmeyanov, L.G. Makarova and I.V. Polovyanyuk, *J. Gen. Chem., 35* (1965) 682.
8 A,D, Lakdawala, M.V. Shirole, S.S. Mandrekar and A.N. Dohadwalla, *Asia Pacific J. Pharmacol., 3* (1988) 91.
9 A.R. Norris, R.Kumar, E. Buncel and A.L. Beauchamp, *J. Inorg. Biochem., 21* (1984) 277.
10 D. Cozak, A. Mardhy, M.J. Olivier and A.L. Beauchamp, *Inorg. Chem., 25* (1986) 2600.
11 A.T. Tu and J.A. Reinosa, *Biochemistry, 5* (1966) 3375.
12 L.M. Twanmoh, H.B. Wood and J.S. Driscoll, *J. Heterocycl. Chem.*, 10 (1973) 187.
13 T.Y. Shen in, *Burger's Medicinal Chemistry*, Part III, 4th Edn., M.E. Wolff, Ed., John Wiley and Sons, New York, 1981, p. 147.
14 T.Y. Shen in, *Advances in Inflammation Research*, Vol. I, G. Weissmann, Ed., Raven Press, New York, 1979, p.535.

**Received: February 1, 1994 - Accepted: March 4, 1994 - Received in camera-ready format: September 13, 1994**

